# Peripheral neuropathy in severe COVID‐19 resolved with therapeutic plasma exchange

**DOI:** 10.1002/ccr3.3397

**Published:** 2020-10-07

**Authors:** Fahad Faqihi, Abdulrahman Alharthy, Ziad A. Memish, Demetrios J. Kutsogiannis, Peter G. Brindley, Dimitrios Karakitsos

**Affiliations:** ^1^ Critical Care Department King Saud Medical City Riyadh Saudi Arabia; ^2^ Research and Innovation Center King Saud Medical City Riyadh Saudi Arabia; ^3^ Department of Critical Care Faculty of Medicine and Dentistry The University of Alberta Edmonton AL Canada; ^4^ Critical Care Department Keck School of Medicine USC Los Angeles CA USA

**Keywords:** COVID‐19, cytokine release syndrome, peripheral neuropathy, therapeutic plasma exchange

## Abstract

Peripheral neuropathies including Guillain‐Barré syndrome may be linked to life‐threatening COVID‐19. Plasma exchange is a safe rescue therapy in severe COVID‐19 with associated neurological manifestations and thromboinflammation.

## Graphical abstract

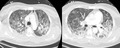

## 1 INTRODUCTION

Peripheral neuropathy (PN) was not linked to severe COVID‐19. We report a previously healthy middle‐age male who had life‐threatening COVID‐19 characterized by PN, acute respiratory distress syndrome, sepsis, and hyperinflammation, all resolved after plasma exchange. Plasma exchange is a safe adjunctive therapy in severe COVID‐19 with neurological manifestations. Brain and peripheral nervous system pathologies were reported in the novel SARS‐CoV‐2 disease (COVID‐19).^1^, ^2^, ^3^, ^4^, ^5^ However, severe COVID‐19 was not previously linked to peripheral neuropathy (PN). Neurological manifestations in COVID‐19 may be partially attributed to the biochemical perturbations of sepsis, neuroinflammation, and cytokine release syndrome (CRS).^6^ Severe COVID‐19 is characterized by acute respiratory distress syndrome (ARDS), sepsis, thromboembolic disease, and CRS.^7^, ^8^, ^9^ We briefly outline a patient with severe COVID‐19 who presented with PN, ARDS, and CRS, all resolved after the administration of therapeutic plasma exchange (TPE) with artificial plasma.

## 2 CASE PRESENTATION

A 44‐year‐old previously healthy man was admitted to the emergency department (ED) on June, 2020, with 12 days of fever (38.3°C), persistent cough, anosmia, diarrhea, myalgias, and progressive bilateral lower limb weakness. The patient mentioned unprotected contact with his brother, who was infected with SARS‐CoV‐2, 1 week prior to the development of his symptoms. Neurological examination showed reduced power (3‐out‐of‐5) in bilateral lower limb muscle groups, plus gait ataxia, and areflexia in bilateral knees and ankles. He had no sensory deficits and no other central nervous system or other neurological signs and symptoms. Emergency electromyography (EMG) of the lower limbs was performed, which revealed delayed latencies but normal conduction velocities (Table 1). Unfortunately, upper limbs were not tested and no biopsy was performed, upon ED admission, as the patient was processed rapidly to investigate the COVID‐19 status. However, these EMG findings were considered to be quasi‐normal, and thus our differential diagnosis included peripheral neuropathy (demyelinating versus axonal) accordingly.^10^, ^11^, ^12^, ^13^ Physical examination depicted bilateral crackles on the lung bases. The saturation of peripheral oxygen (SpO_2_) was 78%, on room air, and the rest of vital signs were within normal limits. The patient was connected to a high‐flow nasal cannula (flow: 60 L/min, fraction of inspired oxygen [FiO_2_]: 40%) maintaining SpO_2_ of 89%. Infection with SARS‐CoV‐2 was suspected due to the epidemiologic background and the clinical presentation.

**Table 1:** Electromyography lower limb findings in our COVID‐19 patient

Lower limb nerves	Distal latency (ms)	Amplitude (mV)	Conduction velocity (m/s)	F waves latency (ms)
Left tibial nerve	(normal ≤ 5.1)	(normal ≥ 4)	(normal ≥ 40)	(normal ≤ 56)
Ankle‐abductor hallucis brevis	6.49	3.45	42	59
Popliteal fossa‐ankle	7.12	3.88	42	57
Right tibial nerve	(normal ≤ 5.1)	(normal ≥ 4)	(normal ≥ 40)	(normal ≤ 56)
Ankle‐abductor hallucis brevis	6.21	4.11	44	55
Popliteal fossa‐ankle	6.34	3.89	43	58
Left peroneal nerve	(normal ≤ 5.5)	(normal ≥ 4)	(normal ≥ 42)	(normal ≤ 56)
Ankle‐extensor digitorum brevis	7.18	4.11	44	52
Below fibula‐ankle	8.22	3.67	45	50
Right peroneal nerve				
Ankle‐extensor digitorum brevis	6.35	3.87	41	60
Below fibula‐ankle	6.78	4.33	44	57

Baseline laboratory results showed lymphocytopenia (0.51 × 10⁹/L, normal: 1.1‐3.2 × 10⁹/L) and increased inflammatory biomarkers defining CRS such as C‐reactive protein (247 mg/L, normal: 0‐5 mg/L), lactate dehydrogenase (1222 µ/L, normal: 100‐190 µ/L), D‐dimers (3.6 mcg/mL, normal: 0‐0.5 mcg/mL), ferritin (1123 ng/mL, normal: 23‐336 ng/mL), and interleukin‐6 (778 pg/mL, normal range 1‐7 pg/mL).^9^ Creatine kinase was slightly increased (616 µ/L, normal: 22‐198 µ/L), but renal function and the rest of biochemistry report were within normal limits. Toxicology screen was negative. The coagulation profile was normal apart from increased D‐dimers. However, low levels of ADAMTS 13 activity with antibody titers within normal limits (TECHNOZYM^®^ELISA) were detected (ADAMTS 13 activity: 8%, normal >10% ADAMTS 13 IgG: 9 µ/L, normal: 6‐12 µ/L).^14^ These tests were performed as the thrombotic risk was considered to be high (D‐dimers > 3 and possible COVID‐19 status).^15^, ^16^, ^17^, ^18^, ^19^, ^20^ Moreover, a full work‐up for other systemic disorders (ie, autoimmune diseases and antiphospholipid antibodies) was performed accordingly.

**Figure 1:**
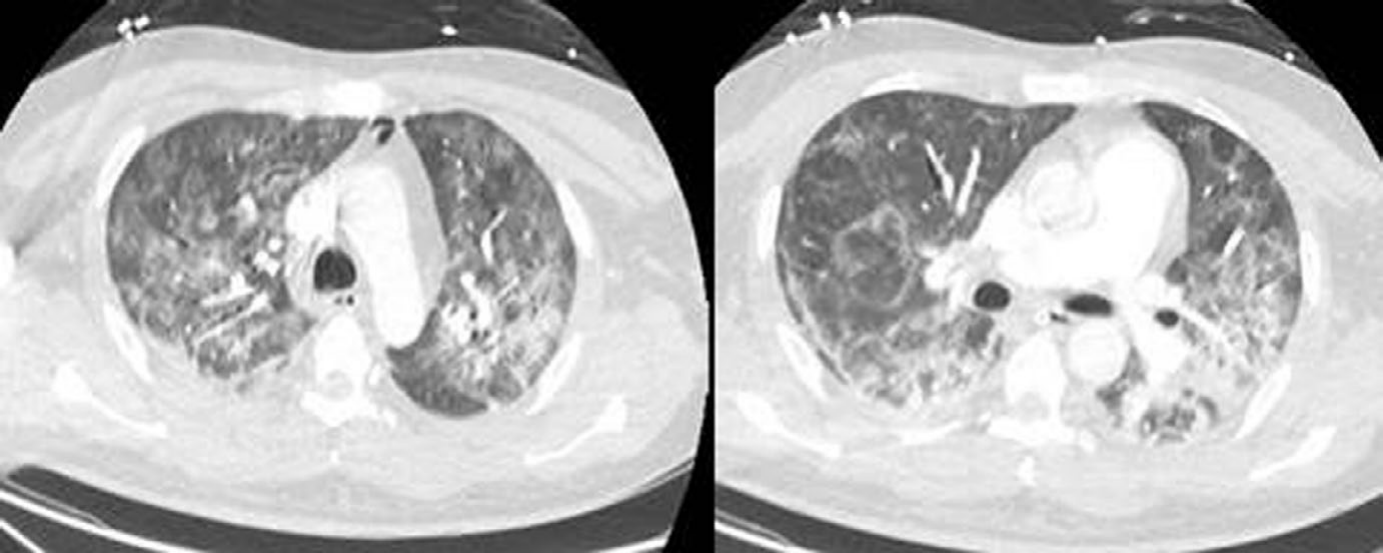
Contrast chest computed tomography scans depicting peripheral bilateral ground‐glass opacities, especially on the right lung, in our patient with severe COVID‐19

Although there were no central nervous system signs or symptoms, a lumbar puncture was performed by a consultant neurologist, and subsequent cerebrospinal fluid analysis revealed a normal cell count (2 × 10⁶/L, normal: 0‐8 × 10⁶/L) and protein (12 mg/dL, normal: 8‐43 mg/dL). SARS‐CoV‐2 infection was confirmed by RT‐PCR assays (targeting for RdRp gene, E gene, and N gene of SARS‐CoV‐2), which were performed on nasopharyngeal swabs, using QuantiNova Probe RT‐PCR kit (Qiagen) in a Light‐Cycler 480 real‐time PCR system (Roche) as previously described.^21^, ^22^ Contrast chest computed tomography scans depicted peripheral bilateral ground‐glass opacities and excluded pulmonary embolism (Figure 1).

Fourteen hours post‐ED admission, the patient developed severe hypoxia (SpO_2_/FiO_2_ ratio: 100) and septic shock; hence, he was intubated and transferred to the intensive care unit (ICU). We administered ARDS‐net and prone positioning ventilation, and empiric therapy with lopinavir/ritonavir, ribavirin, interferon beta‐1b, broad spectrum antibiotics, intravenous vasopressors and hydrocortisone, prophylactic anticoagulation, and supportive ICU care as per hospital protocol.^23^ Echocardiography and cardiac enzymes were normal while lower limb sonography excluded deep vein thrombosis. However, the patient's clinical status was deteriorating; hence, we applied rescue TPE as detailed elsewhere.^9^ Briefly, TPE was initiated using the Spectra Optia™ Apheresis System, which operates with acid‐citrate dextrose anticoagulant as per Kidney Disease Improving Global Outcomes 2019 guidelines.^24^ A dose of 1.5 plasma volumes was used for the first dose then one plasma volume daily. Plasma was replaced with artificial Octaplas LG^®^ (Octapharma AG), which is a fresh frozen pooled plasma product that underwent viral inactivation by prion reduction technology to minimize the possible risk of infectious agents' transmission.^25^ Three daily (4‐hour) sessions of TPE were applied without any complications (ie, allergy, coagulopathy, infections, cardiac, and renal system side‐effects) recorded. After the completion of TPE sessions, SpO_2_/FiO_2_ ratio exceeded 350 with gradual radiological improvement, and we were able to wean off vasopressors. Moreover, patient's inflammatory biomarkers (C‐reactive protein, lactate dehydrogenase, ferritin, D‐dimers, and interleukin‐6 levels) along with the ADAMTS 13 activity/antibody levels were normalized with a parallel sustained increase of lymphocyte counts (Table 2). He was extubated on day‐7 post‐ICU admission. RT‐PCR test and microbiology were negative on day‐18 post‐ICU admission. All the work‐up for other systemic (ie, autoimmune disorders) diseases and viral infections was negative. He was discharged to the neurology ward on day‐20 post‐ICU admission. On day‐30, follow‐up magnetic resonance imaging of head and spine, and motor nerve conduction studies of upper and lower limbs were normal, as was lower limb power and gait. Thereafter, the patient was discharged to home isolation.

**Table 2:** Comparison of parameters before and after three sessions of plasma exchange in our COVID‐19 patient

Parameters	Before TPE	After TPE
PaO_2_/FiO_2_ ratio	100	330
Lymphocyte count (10^9^/L, normal range 1.1‐3.2)	0.51	1.1
C‐reactive protein (mg/L, normal range 0‐5)	247	18
Total bilirubin (μmol/L, normal range 0‐26)	26.2	22.8
Alanine aminotransferase (µ/L, normal range 9‐50)	52	41
Aspartate aminotransferase (µ/L, normal range 15‐40)	39	37
Creatinine (mg/dL, normal range 0.6‐1.2)	1	0.9
Creatine kinase (µ/l, normal range: 22‐198)	616	174
ADAMTS 13 activity (%, normal >10%)	8%	22%
Serum lactate (mmol/L, normal range 1.0‐2.5)	2.9	2.1
Lactate dehydrogenase (µ/L, normal range 100‐190)	1222	185
Ferritin (ng/mL, normal range 23‐336)	1123	382
D‐dimers (mcg/mL, normal values <1)	3.6	0.8
Interleukin‐6 (pg/mL, normal range 1‐7)	778	9.6

Abbreviations: PaO_2_/FiO_2_, partial arterial pressure of oxygen to fractional inspired concentration of oxygen; TPE, therapeutic plasma exchange.

## 3 DISCUSSION

This case report, albeit its many limitations, is shared because the neurological manifestations in severe COVID‐19 are yet to be fully understood. We cannot definitely attribute the putative PN to COVID‐19, as a muscle biopsy and upper lower limb EMG were not performed upon ED admission. However, the lower limbs' EMG findings along with the neurological clinical picture were suggestive of PN.^10^, ^11^, ^12^, ^13^ In a recent large retrospective study, PN was observed in less than 1% of COVID‐19 patients.^26^ We cannot exclude Guillain‐Barré syndrome (GBS) from our differential diagnosis. GBS is an acute immune‐mediated disease of peripheral nerves and nerve roots (polyradiculoneuropathy), which could be triggered by various infections.^27^, ^28^ GBS was recently described in COVID‐19 patients.^2^, ^29^ The typical features of GBS integrate progressive, ascending, symmetrical flaccid limbs paralysis, areflexia, or hyporeflexia, with or without cranial nerve involvement, which can progress over the course of days to several weeks. Although our patient did not have typical manifestations, the possibility of a GBS variant cannot be omitted. Antiganglioside antibodies that are strongly associated with certain forms of GBS were not tested.^27^, ^28^ COVID‐19 appears to have neuroinvasive properties; however, the pathophysiology of immune‐mediated peripheral neuropathy remains obscure. Molecular mimicry, which is an important mechanism in creating autoimmune disorders, may have a role in the development of COVID‐19‐associated GBS.^27^, ^28^ COVID‐19‐related hyperinflammation and immune system dysregulation, which in turn could generate autoimmune processes, could be another potential mechanism.^6^, ^7^, ^8^, ^9^

In this report, whether the application of TPE accounted for the neurological improvement of the patient is unclear; however, given the severity of our patient's clinical picture, we speculate that it might have helped, especially given its biochemical plausibility. TPE can remove interleukins‐3, 6, 8, 10, interferon‐gamma, tumor necrosis factor‐alpha, and various immunoglobulins of the IgG class.^9^, ^30^, ^31^ Moreover, the main rationale for applying TPE on COVID‐19 is the suppression of the thromboinflammation and the amelioration of the ensuing microangiopathy.^15^, ^16^, ^17^, ^18^, ^19^, ^20^ In our patient, we observed normalization of inflammatory biomarkers and sustained increase in lymphocyte counts and ADAMTS 13 activity after three sessions of TPE. Elevated levels of inflammatory biomarkers and lymphocytopenia are predictors of severe COVID‐19 and death while low levels of ADAMTS 13 were correlated to poor prognosis in patients with sepsis and multi‐organ failure.^7^, ^8^, ^9^, ^32^ Notably, extracorporeal blood purification therapies were previously used in severe sepsis and in COVID‐19 with associated CRS.^9^, ^30^, ^31^ Also, no specific antiviral therapies for COVID‐19 were approved for widespread use thus far. We used TPE with artificial plasma replacement for the first time, to our knowledge, to treat severe COVID‐19 with neurological manifestations and associated CRS. Also, we documented a thromboinflammation profile in severe COVID‐19, which exhibited similarities to sepsis and other systemic thrombotic microangiopathies, although no severe coagulopathy was evident in our patient.^33^, ^34^, ^35^ The hyperinflammation response and microthrombosis in COVID‐19 result in multi‐system organ failure with fatal outcomes.^15^, ^16^, ^17^, ^18^, ^19^, ^20^ SARS‐CoV‐2 has a versatile organotropism for extra‐pulmonary targets; moreover, it can bind to the ACE‐2 receptor facilitating endothelial injury and thromboinflammation, on the grounds of dysregulated renin‐angiotensin‐aldosterone and immune systems' responses.^36^

This case report has limitations, which prevent its generalizability. Apart from TPE, the patient received empiric therapies and ICU supportive care. We are uncertain of their effects on inflammatory mediators or the extent to which these therapies affected survival or improved the neurological manifestations. The effect of TPE upon SARS‐CoV‐2 shedding is also unclear. Moreover, the natural course of SARS‐CoV‐2 viremia is still undetermined as reinfections and/or recurrently positive RT‐PCR results were described.^37^, ^38^, ^39^ Hence, the optimal TPE regime remains to be further clarified in future studies. Nevertheless, we are encouraged that prompt initiation of TPE was associated with resolution of CRS and amelioration of the clinical picture in our patient with severe COVID‐19.^9^, ^40^ Previous studies advocated that, at this stage of the disease, mitigating the dysregulated immune and inflammatory response could be more important than targeting the virus per se.^41^ Also, unlike several immunomodulatory therapies, there is minimal immunosuppression associated with plasma exchange. However, the cost and resources of TPE should be carefully evaluated when the latter is employed in the management of COVID‐19. Hence, we are suggesting its use only as an adjunctive rescue therapy in patients with life‐threatening disease.^9^

## 4 CONCLUSION

The correlation between severe brain pathology and COVID‐19 was previously established. However, the putative link of peripheral neuropathies including Guillain‐Barré syndrome to life‐threatening COVID‐19 needs to be further elucidated. Plasma exchange is a safe rescue therapy in life‐threatening COVID‐19 with associated neurological manifestations and thromboinflammation.

## ACKNOWLEDGEMENTS

Published with written consent of the patient.

## CONFLICT OF INTEREST

None declared.

## AUTHORS CONTRIBUTION

FF, AA, and DK: treated the patient and drafted equally the manuscript. ZAM, DJK, and PGB: provided expert consultation regarding the neurological findings, and equally drafted and reviewed the manuscript. The final version of the manuscript was reviewed and approved by all authors.

## ETHICS APPROVAL AND CONSENT TO PARTICIPATE

The study was approved by the Institutional Review Board of King Saud Medical City, Riyadh, Kingdom of Saudi Arabia, protocol/serial number: H‐01‐R‐053, IORG0010374, H1R1‐29‐Apr20‐01. Written informed consent was obtained from the patient. The study is also registered at ISRCTN (ISRCTN21363594; doi.10.1186/ ISRCTN21363594).

## CONSENT FOR PUBLICATION

Written informed consent was obtained from the patient for publication of this study. A copy of the written consent is available for review by the Editor‐in‐Chief of this journal.

## Data Availability

The datasets used and/or analyzed during the current study are available from the corresponding author on reasonable request.
